# In vitro fertilisation mix-ups and contested parenthood

**DOI:** 10.1136/jme-2025-111285

**Published:** 2026-01-09

**Authors:** Sinead Prince, Andrew John McGee, Hilary Bowman-Smart, Julian Savulescu

**Affiliations:** 1 Centre for Biomedical Ethics, National University of Singapore Yong Loo Lin School of Medicine, Singapore; 2 Australian Centre for Health Law Research, Queensland University of Technology, Brisbane, Queensland, Australia; 3 University of South Australia, Adelaide, South Australia, Australia; 4 Oxford Uehiro Institute, University of Oxford, Oxford, UK; 5 Murdoch Children’s Research Institute, Melbourne, Victoria, Australia

**Keywords:** Ethics- Medical, Fertilization in Vitro, Philosophy- Medical, Reproductive Medicine, Women

## Abstract

In 2025, an Australian couple asked to have their remaining embryos moved to another clinic, only to discover that the child they had birthed 2 years earlier had not come from their own embryos, but an embryo belonging to a different couple. These situations can lead to disputes about who is recognised as ‘the parents’ in the biological or social sense, as well as who has moral or legal claims to parental rights and responsibilities. In terms of specific legal disputes over custody or guardianship, the matter will generally be resolved in the best interests of the child. However, one of the considerations relevant to this child’s best interests is the question of biological relatedness, even if only due to the social weight it is often granted. This paper will argue that the current presumption in favour of genetics as determinative of biological relatedness is rebuttable in favour of the gestational relationship. Furthermore, there are other reasons to give weight to the moral, legal, or social claims of the gestational progenitors, such as bonds with the infant that have already been developed. However, such mix-ups will happen again and, in light of genomic technologies, may be discovered in vivo or immediately after birth, in which the courts may be ill-suited to determining the best interests. As such, legislative approaches to resolving parenthood in such cases must be proactively developed.

## Introduction

 In 2023, a woman in Australia gave birth to a child through in vitro fertilisation (IVF) that was, unbeknownst to anyone involved, not genetically related to her or her partner. The mistake was discovered in February 2025, when the woman and her partner (hereafter referred to as the ‘gestational progenitors’^1^) requested their remaining embryos be moved to a different clinic.^[1]^ It is yet unknown what the genetic progenitors intend to do. However, if a legal dispute about parentage arises, a decision will have to be made as to which parents have the strongest claim to legal parenthood over the child. In IVF mix-ups,^[2]^ there are three key practical questions that arise. First, what constitutes biological relatedness in these cases, and is it broader than mere genetic relatedness? Second, what is the normative significance of the various kinds of biological relatedness in this IVF mix-up case? And third, all things considered, who should be permitted to rear the child?

When it comes to the idea of ‘parenthood’, we must start by acknowledging that there are a number of related but distinct questions at hand. This is in part because we use the term ‘parenthood’ in multiple different contexts—biological, social, moral, and legal—with a concomitant range of meanings. First, in these cases, we might consider the more abstract or conceptual question around who counts as a ‘biological’ parent and what constitutes biological relatedness. For the purposes of clarity, when referring to *biological relations*, we will use the term ‘progenitors’ rather than ‘parents’. Second, there is a moral or normative question around who has rights, duties, or responsibilities in terms of the parent-child relationship; this is connected to the debate on the normative significance of biological relatedness and the importance of the social role of parent. Third, there are legal decisions that must be made, in terms of legal status (eg, names on birth certificates), legal obligations (eg, child support) and day-to-day matters of custody and guardianship. Fourth, there is also a social element—who is ‘recognised’ as a parent in terms socially and who fulfils the social role of parent (who may be neither a biological nor legal parent/guardian, eg, a step-parent or family friend).

Generally, most jurisdictions have a statutory legal presumption that the person who births the child is the legal parent by default. Legal positions diverge when disputes arise; while most courts adopt a best interests approach,^2^ taking into consideration the child’s mental, physical, social, and relational well-being, jurisdictions weigh the role of genetic relatedness as contributing to the child’s best interests differently. For the purposes of this paper, we will assume that both the gestational carriers and genetic progenitors would love the child and promote the child’s overall welfare. In doing so, we can isolate the role of biological relatedness in determining legal parenthood. This is also important in cases when the mistake is discovered in vivo and the child’s best interests approach (which applies from birth) may fail to provide determinative reasons about legal parenthood. This brings us back to the first and second questions, what counts as biological relatedness, and when all else is equal, what is the significance of biological relatedness between ‘parent’ and child?

As such, in this paper, we consider what constitutes biological relatedness between each set of progenitors and the child—since the concepts of genetic and biological relatedness are not co-extensive—and the normative significance of these in determining which progenitors ought to be permitted to rear the child in this IVF mix-up. We will first examine how the current legal approaches consider the role of genetic relatedness in determining parenthood. We will then consider the reasons why genetic and gestational relatedness might have normative significance in determining which parents’ ought to be permitted to rear the child. We question the widely held premise that the child in question is ‘not related’ to the gestational carrier—a ‘stranger’s baby’^3–5^—and as such, that it is not in the child’s best interests to be placed with the gestational progenitors. We will ultimately argue that, in this IVF mix-up, the gestational relationship can be more normatively significant than the genetic one.

## What does the law say?

In Australian law, the gestational progenitor is the presumed legal parent of the child.^[3]^ When the child is genetically unrelated to the gestational progenitor, genetic progenitors are likely to succeed in a transfer of legal parenthood *if* a lawful surrogate agreement is in place.^[4]^ A surrogacy agreement is a legal document that affirms the intention of the surrogate to relinquish legal parenthood *and* guardianship to the intended parent(s) on birth. However, this IVF mix-up is both legally and conceptually distinct. No surrogate agreement was in place because there was never an intention for any party to enter a surrogacy arrangement; the gestational progenitors expected to implant one of their own embryos.^[5]^ This is one reason why, legally, there is always a presumption, although in some cases rebuttable, that the gestational carrier is the legal parent. The genetic progenitors might therefore challenge the application of surrogacy laws because they did not consent to the implantation of their embryo in the gestational progenitor.^[6]^ Similarly, the gestational progenitor might challenge the applicability of these laws on the grounds that they did not consent to the implantation of *another couple’s embryo*, even if they did consent to the implantation process. Conceptually, a surrogate requires agreement between all parties; otherwise, rape would also be a form of surrogacy. Relying on the lexical priority afforded genetic progenitors in surrogacy laws would therefore be unjustified. Nevertheless, if the genetic progenitors challenge the legal presumption of parenthood, the courts will likely determine legal parenthood according to the best interests of the child.^[7]^ Doing so requires considerations of: the child’s care, welfare, and development; how long the child has been living with whom; capacity to raise the child; and any empirical evidence as to the importance of genetic or biological relatedness in the child’s development and psychological well-being.

In the USA, while the gestational progenitor is the presumed legal parent, the law has been interpreted differently in custody battles and reproductive disputes with priority afforded to the genetic relationship.^6–10^ In 2000, an IVF mix-up resulted in the gestational progenitors voluntarily relinquishing a child to the genetic progenitors, though in this case, the gestational progenitors had twins, of which only one was genetically related, leaving the gestational progenitors with one child.^[8]^ Interestingly, the court dismissed the genetic progenitors’ claims that the gestational progenitors were ‘genetic strangers’ to the child and held that current reproductive technologies undermined the strength of genetics to determine the ‘natural parents’. Nevertheless, the court adopted an intent-based approach and concluded that the genetic progenitors were the ‘natural parents’ insofar as they were the ones who ‘purposefully arranged for their genetic material’ to create their own child to raise.^[9]^ This is in line with accounts of parenthood that prioritise causality—that is, who caused the child to come into existence.^11^ In all cases since, US courts have ruled in favour of the genetic progenitors.^8^ For example, in 2019, a couple was forced to relinquish custody and legal parenthood of two children to each of the child’s respective genetic progenitors.^[10]^ In a 2021 lawsuit,^[11]^ two couples in an IVF mix-up agreed to swap the infants, returning each child to their genetically affiliated parents.^12 13^

In Israel, a similar IVF mix-up came before the Supreme Court in 2025, in which the court awarded legal parenthood and custody to the gestational progenitors.^14^ Although the mistake was discovered during the pregnancy, the genetic progenitors were not known until sometime after birth. The child suffered from a heart defect resulting in the gestational progenitors caring for her during multiple life-saving cardiac interventions. Although the court acknowledged there were no laws to determine legal parenthood in IVF mix-up cases, the majority opinion, written by Justice Yael Willner, drew from existing egg donation and surrogacy laws that default legal parenthood to the gestational progenitor. They reinforced this decision by considering the child’s best interest; professional evaluations of the child’s welfare found that the child had lived with and formed deep attachments with the gestational progenitors and had complex medical needs that might be jeopardised by a transfer. Dissenting Justice Daphne Barak-Erez adopted the intentional approach and held that the genetic progenitors should have legal recognition and noted that neither the genetic progenitors nor the gestational carrier had acted inappropriately.^[12]^ The court nevertheless acknowledged that the child should maintain a connection with their genetic progenitors but did not confer any legal parenting responsibility, only ordering welfare authorities to develop a plan for maintaining the relationship between the child and the genetic progenitors. The court also agreed that such matters should be proactively addressed by the legislature, particularly considering the increasing power and prevalence of assisted reproductive technologies.

These different legal approaches rest on valid, though potentially conflicting, intuitions about parenthood: surely those who create an embryo have a claim to be recognised as the legal parents of the subsequent child that they intended to have. Yet, just as surely, those who undergo implantation, pregnancy, childbirth, and social parenthood also have a similar claim to the child they have gestated and for whom they have cared. We shall explore the normative reasons behind these claims in more detail.

## How can we think about biological relatedness in IVF mix-up cases?

Biological parenthood is often described or implicitly understood in terms of *genetics*—the ‘genocentric’ view.^15^ A range of definitions of what constitutes genetic parenthood or progeneration has been put forward in the literature; however, scenarios arising from novel reproductive technologies (eg, mitochondrial replacement, in vitro gametogenesis) create challenges for each of these.^16^ Other theorists, such as Gheaus, have put forward the position that it is the gestational progenitor who should be considered a, or the, ‘biological parent’.^17^ In philosophy, there are a range of views on the normative relevance of biological relation to moral parenthood, namely, the possession or conferral of parental rights, obligations, and responsibilities.^17 18^ Some views of moral parenthood, for example, Millum,^19^ emphasise investment or labour, whereas others, for example, Ferracioli,^20^ view it as a commitment to the flourishing of the child.

Rather than lay out a particular framework of the normative significance of biological relatedness, in this section, we will undertake a comparative analysis of both the gestational and genetic relationships in this IVF mix-up. We will consider the biological nature of each relationship, whether the biological relationship can be separated from the relational one (if at all), the role of intention in the relationship, and the impact of each relationship on the child’s well-being. As such, we will consider both the intrinsic and instrumental value of each biological relationship in this particular IVF mix-up, as well as other reasons to give weight to the moral, legal, or social claims of the gestational parents/progenitors, such as existing bonds with the infant and the impacts of biological relationships on reproductive autonomy.

### In favour of the genetic progenitors

The fact that the genetic progenitors are biologically related to the child is undeniable; but for the parents harvesting *their* gametes through the IVF process, that specific child would not exist. The genetic progenitors created the child’s genetic makeup, the impact of which lasts the child’s entire life. The value placed on genetic relationality is also significant, particularly because biological relatedness is commonly understood in terms of genetics. There are a range of reasons why many societies view biological relations—primarily understood as genetic relations—as meaningful or important, particularly in terms of kinship structures, parental duties and personal identity.^21^ Our personal and psychological identities are often wrapped up with our genetics, kinship, and heritage, and as such, the answers to questions such as who we are, who we belong to, and where we come from become complicated when we cannot genetically relate with the relational unit to which we socially and structurally belong.^22^ For this reason, the Singaporean Court of Appeal established that the gestational progenitors of children with genetic relations that were unintended in IVF mix-up cases could sue for ‘loss of genetic affinity’.^[13]^ Therefore, we might consider prioritising the genetic progenitors on the basis that genetic relations are strongly *perceived* as conceptually and normatively important in parent-child relationships.

However, some research suggests that the need for genetic relatedness within ‘nuclear’ families is based on heteronormative expectations that a family consists of mother, father and genetic offspring.^23^ A ‘bionormative’ approach constructs parenthood as private (the state is not involved), exclusive (it may not be usurped by anyone else), and binary (ie, only two parents).^24^ However, family units are often diverse and blended, with stepparents, single parents, same-sex parents, or adopted parents. Some philosophers view the value of genetic relatedness as pernicious insofar as it excuses unjustified favouritism and causes unjust social and economic inequality.^25^ Some claim that our beliefs in the value of those genetically related to us have obvious evolutionary selective advantages and thus no independent moral worth.^26[14]^ While some research suggests that adopted children experience a greater risk of psychological and behavioural difficulties, this is being increasingly attributed to the context surrounding the adoption rather than the lack of genetic relatedness.^27^ Furthermore, the quality of the parent-child relationship is a well-established protective factor in the child’s mental health outcomes.^28^ The exact instrumental value of genetic relatedness in protecting or enhancing child psychological well-being is, at least in part, an empirical question and therefore outside the scope of this paper. We can nevertheless accept that genetic relatedness has at least *some* normative weight in parent-child relationships.

The relative strength of this normative value, however, is contextual. After all, children can be lovingly adopted, genetic progenitors can be restricted from visiting or raising their child, children are legitimately born through donor conception to loving non-genetically related persons, and sometimes a gestational progenitor might not inform the genetic father of the child’s existence. The quality and context of the interparental and parent-child relationships are relevant for determining moral parenthood. This is not to say that genetic relatedness is irrelevant or unimportant; indeed, assisted reproductive technology legislative frameworks have rejected donor anonymity following the modern right to know one’s genetic heritage,^[15]^ and the (Australian) foster system generally prioritises returning the child to their genetic progenitors when possible.^29^ Genetic relatedness is clearly important in determining moral parenthood (and in several jurisdictions, legal parenthood), but the view that genetic relatedness is a *necessary* component of moral or legal parenthood is a rebuttable presumption, if it is one at all. Genetic relatedness is relevant, but it is not always the closest genetic relation that has the strongest claim to moral or confer legal parenthood, particularly when someone else with biological relatedness to the child might be more suited to raising the child (the latter we will consider in the next section).

A response to this, and a second reason why we might give greater weight to the genetic relationship in this case is that the genetic progenitors *intended* to have, and thus invested in having, a child from IVF; they went through the physical, financial, and emotional stress of IVF to have a child who was genetically related to them. Unlike situations in which children enter foster homes or are cared for by other relatives due to parental incompetence or an unfortunate inability to care for a child, the genetic progenitors in this case intentionally and desperately wanted a child to whom they were genetically related. Indeed, it is likely that the genetic progenitors may suffer at least psychological harm in being denied parental rights to their genetic child. These arguments might also apply to the gestational progenitors who, by virtue of undergoing IVF, equally intended to have a child that was genetically related to them—namely, not the child in question.

In cases of symmetrical intentionality, a possible resolution is that it is better for at least one couple to satisfy their reproductive intentions (as evidenced by using IVF), than for neither couple to satisfy their reproductive intention to have a child genetically related to them.^[16]^ However, we cannot reduce the concept of reproductive intent for using IVF to such a narrow definition: there are many intentions for using IVF including to have a child (as distinct from the genetic traits of the child), such as to experience gestation and childbirth and to have a family. Indeed, can we make such a claim that the intention to have genetically related offspring is more valuable than the satisfaction of other reasons to use IVF, such as to experience gestation, to become a parent, or to simply have a child at all? Even if the intention to have genetically related children was originally quite significant to each couple, we ought not to presume that the gestational progenitors maintained such intentions after conceiving and gestating the child: gestation is a transformative experience, and the gestational progenitors may legitimately hold different views on the value of genetic affinity post-gestation.^30^

There are some good reasons to endorse privileging the genetic relationship in this IVF mix-up. There is the pragmatic observation that genetic relations have significant social and personal value, and the genetic progenitors clearly intended to have the child in question (having invested heavily through IVF). However, a desire to rear a particular child is not a sufficient reason for allowing them to do so. In this case, such desires must still be balanced against what is best for the child,^[17]^ as well as the normative and conceptual relevance of the relationship between the gestational progenitors and the child.

### In favour of the gestational progenitors

Running parallel to the genetic progenitors’ claim to the child is the claim by the gestational progenitor. There are several elements to the gestational claim: first, the case for gestation as being conceptually relevant to biological relatedness; second, the social and relational significance of the gestational relationship, particularly to the gestational progenitor and the child; and, finally, the moral significance of favouring the gestational relationship to protect pregnant people’s autonomy.

We will begin by providing a brief overview of reasons to consider gestation as conceptually significant for our understanding of biological relatedness, in contrast to the ‘genocentric’ view.^15^ To put it simply, but for the embryo’s gestation in the gestator’s body, which enabled the embryo to grow by providing all the resources it needed, the fetus would not have developed into a person. While the genetic progenitors provide DNA ‘instructions’, the gestational progenitor provides the organic material to gestate the fetus, namely, the proteins, hormones, minerals, amino acids, sugars, and materials that physically develop the fetus into an infant. Although the same embryo *could* have been implanted in the uterus of a genetic progenitor (hereafter the ‘genetic mother’), there is no guarantee this would be *successful* in resulting in a live birth. That is to say, except for the contribution of the gestational progenitor, *a* future child from that particular embryo may not have come about. As such, gestation has a significant contribution to the *kind* of person that comes about—that is, a qualitative impact on the personal identity of the future child.

Even if the embryo was successfully implanted, gestated and birthed by the genetic mother, the provision of these other materials means that the fetus would differ qualitatively, although no doubt in subtle ways. For example, we could imagine that the genetic mother had severe maternal iodine or folate deficiencies she was unaware of, which would cause neural tube defects in the child that would not be caused if the actual gestational progenitor had no such maternal iodine or folate deficiencies. Thus, the gestational progenitor can provide a significant contribution to the traits and features of the future child, which includes shaping the genetic and phenotypic makeup of the child. This is because pregnancy is not merely the independent development of a fetus inside a womb to infancy; gestation involves maternally caused epigenetic changes to the fetal DNA.^31–33^ Research shows that the maternal environment can cause long-term impacts on the child’s health, psychology, and lifespan. The gestational progenitor’s microbiome,^34^ immune systems,^35^ stress levels,^36^ and general health have positive and negative long-term impacts on the child’s epigenetic makeup and subsequent development, all of which can shape the child’s identity. The maternal role in *mediating* the fetal environment is essential to the child’s DNA and development. Furthermore, during pregnancy, bidirectional DNA exchanges occur that result in microchimerisms in both the gestational progenitor and fetus.^37 38^ Recent research also indicates that pregnancy causes neuroanatomical changes to the mother’s brain.^39^ The gestational progenitor thus also contributes the kind of material thought to be only provided by the genetic progenitors: the genetic progenitors might have written the code, but the gestational carrier actually brought the child to life with her own body. The fact that the gestational progenitor gestated the infant in question presents a unique biological relationship between the gestational progenitor and the existing infant: the bond is not a hypothetical potential but factual reality. The gestational mother and infant were biologically embodied. While there is physical, emotional, and psychological labour in egg retrieval for IVF, this pales in comparison to the physical, emotional, and psychological labour invested by the gestational progenitor during pregnancy and childbirth. The gestational progenitor is objectively biologically related to the child (through both genetics and biological development). An argument could be made that these relations encompass more biological relatedness, in both variety (genetic and organic) and in quantity (epigenetics and embodiment) than those between the genetic progenitors and the child. Nevertheless, the fact that the gestational progenitor has biological ties to the fetus renders the conceptual line between biological relatedness and unrelatedness blurry. As such, the normative significance of biological relatedness can be achieved without genetic progenitors.

Second, the gestational relationship inherently creates *relational* relatedness, particularly if the gestational progenitor believes the child is genetically related to her, as was the case in these embryo mix-ups. Throughout the pregnancy, birth and potential breastfeeding, the gestational progenitor can fulfil an important and unparalleled social role in caring, loving, and protecting the fetus or infant. Indeed, in this case, the gestational progenitor has (presumably) undertaken IVF to become pregnant for a child she wants to raise.^40^ Creating a human from implantation through 10 months of pregnancy, and undergoing the difficult and life-threatening experience of childbirth, is not merely a biological experience, but also a phenomenological one.^30^ Qualitative studies with women have found that experiencing pregnancy is a fundamental and irreducible experience, one that transforms the very definition of the woman’s perception of the self and the world around her.^30^ This is uniquely so for women of colour, women with disabilities, and women living through pandemics or experiencing high-risk pregnancies.^41–44^ Unlike a genetic progenitor, a gestational progenitor *necessarily* experiences a phenomenological transformation as part of their biological relationship with the fetus. That is, the strength of the biological relationship between a gestational progenitor and a child is *inherently* relational. Genetic relatedness lacks such equivalent intrinsic relationality.

Third, research suggests that the maternal-infant bond is not merely relational, but essential to the child’s emotional, behavioural, cognitive, and neurobiological well-being.^45^ This bond begins *during* pregnancy; research suggests that the quality of this bond affects the child’s psychosocial well-being insofar as the quality of maternal prenatal attachment mediates or protects the quality of the childbirth experience and postnatal attachment bond.^46^ This bond also strengthens *after* childbirth. Attachment theory, as developed by Bowlby^47^ and Ainsworth,^48^ suggests that the emotional bond between an infant and their caregivers in the first two to three years of life is essential to shaping the child’s future sense of security, social development, capacity for emotional regulation, and resilience. According to attachment theory research, separating an infant from their mother results in distress responses, disrupted attachment formation and long-term difficulties in emotional regulation, trust in relationships, and vulnerability to depression and anxiety in later life.^49 50^ In circumstances where the gestational progenitor continues to provide care to the child after birth, protecting this relationship becomes critical to the formation of the child’s well-being. This empirically established and significant attachment between gestational progenitor and child is (unfortunately) simply *unparalleled* by the genetic progenitors with the child.

Finally, if the genetic progenitors are permitted to rear the child, the gestational progenitor will become an unconsenting surrogate.^51^ The premise under which they consented to pregnancy for their own purposes is fundamentally different from that if they must relinquish the child; the relevant facts to a person agreeing to become pregnant necessarily include whether that person will rear the child. Insofar as we cannot proactively force people to be surrogates for children (ie, rape), we cannot retroactively do so. For example, if we are justified in forcing a gestational progenitor to relinquish a child to satisfy another’s reproductive desires, such a policy might also justify forcing a gestational progenitor to continue the pregnancy of a fetus after they have mistakenly or wrongfully been impregnated. The nature of this situation is that such policies, as in place in the USA, would justify the grave physical violation of people’s reproductive autonomy. While the genetic progenitors would become unconsenting embryo donors if the gestational progenitors were permitted to rear the child, insofar as there is simply no other way for a child to gestate without a mutually embodied physical, emotional, and psychological relationship between gestational progenitor and child, there is no equivalent biological relationship between the gestational progenitors and the child, all else equal.

## Moving Forward

We have argued that despite the obvious normative relevance of genetic relatedness, if all else is equal in this IVF mix-up case, the gestational progenitors should be permitted to rear the child. As such, if a legal dispute arises and the best interests approach is applied, insofar as the courts consider biological relatedness, they should take into account the strength of the gestational relationships, as well as the existing bonds between the infant and the need to protect the progenitors’ reproductive autonomy.

Nevertheless, cases such as these will inevitably continue to arise for courts to resolve and may prevent different factors that affect how we consider biological relatedness. There are several ways we might anticipate these situations arising and create options for a more expansive understanding of parenthood.

One option is to separate legal parenthood from custody or child-rearing agreements. For example, the Saudi Arabian government bought neighbouring houses for parents who had mistakenly been raising each other’s child for 4 years.^52^ The Israeli Supreme Court also made special orders, namely, ordering welfare authorities to develop a plan for maintaining the relationship between the child and the genetic progenitors, despite ordering legal parenthood to the gestational progenitors.^14^

Another option is to reconceptualise parenthood and recognise multiple parentage. Several jurisdictions have also begun recognising multiple parentage in a variety of ways (eg, the USA, the UK, New Zealand, and Canada) to recognise the multiple roles of adults in LGBTQ families, the people involved in assisted reproductive technologies, as well as the variety of family make-ups arising in divorce, co-parenting, and cohabitation agreements and stepfamilies.^53–55[18]^ However, whether multiple parentage laws ought to have implications for guardianship or custody orders remains disputed and open to future research.^53 56 57^

Our claims that the gestational progenitors ought to be entitled to rear the child apply strictly to this IVF mix-up and similar cases. In a new era of increasing use of increasingly accurate and differential assisted reproductive technologies, we ought to proactively develop better legal frameworks and social structures for dealing with likely more frequent disputes and evolving technologies that challenge our traditional understandings of parenthood.

## Data Availability

There are no data in this work.

## Footnotes

For the simplicity of scope, this paper refers to cases when either the pregnant person or both the pregnant person and their partner are claiming legal parenthood to the child. We do not apply our reasoning to cases when the pregnant person’s partner is making a legal claim on their own accord.

For the purposes of this paper, we use ‘IVF mix-up’ to refer to cases in which the child born is genetically unrelated to the gestational progenitor and their partner.

See, for example, the *Status of Children Act 1978* (Qld), which indicates that the woman who gives birth to the child is the lawful mother of the child and her husband or civil partner is the presumed father of the child.

Provided the steps in relevant legislation are complied with: See *Surrogacy Act 2010*. Of course, there was no intention here for any surrogacy, still less for a surrogacy agreement: each woman wanted to carry her own child. See text.

It might be tempting to say that she is therefore an unconsenting surrogate because she carried an embryo of another couple to term, but calling her an ‘unconsenting surrogate’ begs a crucial question, as we shall see. For now, though, we note only that, without any surrogacy or other agreement, the gestational progenitor is presumed to be the lawful parent of the child.

See, for example, *Assisted Reproductive Technology Act 2024* (Qld) s 16, which requires the written consent of the person.

See, for example, *Family Law Act 1975* (Cth) S 60CA.

See, for example, *Perry-Rogers v*. Fasano, 2000, 715N.Y.S.2d 19. Supreme Court of New York, Appellate Division.

This approach was developed in *Johnson v Calvert,* 1993, No. S023721, Supreme Court of California.

See, for example, *Manukyan v. CHA Fertility*, 2019, 19STCV23892, Superior Court of the State of California, County of Los Angeles.

See, for example, *Cardinale and Cardinale v California Center for Reproductive Health*, *et al*, Complaint lodged in the Superior Court of the State of California, County of Los Angeles.

Namely, the mistake arose entirely from the IVF clinic’s negligence.

See, for example, *ABC v Thomson Medical Pte Ltd* [2017] SGCA 20. The court placed particular emphasis on the Indian ethnicity of the child who was born to a German Chinese couple seeking IVF for a genetically related child.

Fitz Patrick does not hold this view himself but is explaining it for the purposes of the article.

See, for example, the National Health and Medical Research Council’s ‘Ethical guidelines on assisted reproductive technology in clinical practice and research’ (2017), which has required clinics to only use samples from donors who consent to the release of identifying information since 2004.

In this IVF mix-up, there is only one child whose legal parenthood is in dispute. However, in some IVF mix-ups, there are two children in question, and a resolution might be that the children could be ‘swapped’, as has occurred in some cases in the USA. However, there are limitations to this resolution’s applicability. First, this might only apply to cases when the mistake is discovered prior to birth, and no external attachment or bond has formed between parents and child. Second, even then, experiencing gestation and the gestational bond between gestational carrier and child may be too strong to consider ‘swapping’ as an ideal solution.

One interesting circumstance might be when the genetic progenitors, due to the mistake, no longer have any stored embryos or cannot undergo further IVF to have another child. Does this change anything? One response might be that the lost opportunity to ever raise a child to whom the parents are genetically related due to the negligent mistake of the IVF clinic is a tragic consequence and, indeed, arguably worse than the lost opportunity to have the child in question. This claim might also be particularly strong if the gestational progenitor has other children or has more embryos that she could use to have her own genetically related child. However, it seems strange to argue that the parent that has the strongest normative reasons to moral parenthood, and thus legal guardianship, depends on whether the parents could be essentially compensated by already having or conceiving other children. Whether a person has a normative claim to legal parenthood of a child is not a question of whether it is fair to the parent to distribute the child in so and so a way but should be whether this relationship is in the child’s best interests. Nevertheless, the presence of alternative options for having children may have some normative relevance. It might be relevant as a tiebreaker, when all the other normative considerations haven’t yielded a decision.

For example, California, more than two parents can be legally recognised in the best interests of the child: Cal. Fam. Code § 7612(c). Similarly, in Canada, some provinces permit recognition of more than two legal parents; British Columbia *Family Law Act (2014*) recognises more than two parents. While the UK does not permit more than two legal parents, more than two people can share parental responsibilities through court orders in certain arrangements, such as step-parents, guardians or intended parents through co-parenting. See, for example, X & Anor v. B & Anor [2022] EWFC 129, in which two loving, platonic partners sought parenting responsibility for a child. Similarly, in Australia, while more than parents cannot be legally recognised as parents, shared parental responsibility can be awarded by the courts.
